# Effect of artificial cycle with or without GnRH-a pretreatment on pregnancy and neonatal outcomes in women with PCOS after frozen embryo transfer: a propensity score matching study

**DOI:** 10.1186/s12958-022-00929-y

**Published:** 2022-03-25

**Authors:** Yue Wang, Wen-Hui Hu, Qi Wan, Tian Li, Yue Qian, Ming-Xing Chen, Xiao-Jun Tang, Qian Feng, Xiang-Qian Meng, Enoch Appiah Adu-Gyamfi, Yu-Bin Ding, Li-Hong Geng, Xing-Yu Lv, Zhao-Hui Zhong

**Affiliations:** 1 Department of Epidemiology, School of Public Health and Management, Research Center for Medicine and Social Development, Innovation Center for Social Risk Governance in Health, Chongqing Medical University, Chongqing, 400016 China; 2 Xinan Gynecological Hospital, Chengdu, 610011 China; 3 Department of Gynecology and Obstetrics, West China Second Hospital, Sichuan University, Chengdu, China; 4 Key Laboratory of Birth Defects and Related Diseases of Women and Children (Sichuan University), Ministry of Education, Chengdu, 610041 China; 5 The Department of Reproductive Medicine, the First Affiliated Hospital of Chongqing Medical University, Chongqing, 400016 People’s Republic of China; 6 Department of Epidemiology, School of Public Health and Management, Research Center for Medicine and Social Development, Chongqing Medical University, Chongqing, 400016 China; 7 Joint International Research Laboratory of Reproduction and Development of the Ministry of Education of China, School of Public Health and Management, Chongqing Medical University, Chongqing, 400016 China; 8 Chongqing Hospital of Traditional Chinese Medicine, Chongqing, 400000 China

**Keywords:** Frozen embryo transfer, Polycystic ovary syndrome, artificial cycle, Gonadotropin-releasing hormone agonist, Propensity score matching

## Abstract

**Background:**

In frozen embryo transfer (FET), there is limited consensus on the best means of endometrial preparation in terms of the reproductive outcomes in women with polycystic ovary syndrome (PCOS). The present study aimed to compare the pregnancy and neonatal outcomes following artificial cycle FET (AC-FET) with or without gonadotropin-releasing hormone agonist (GnRH-a) pretreatment among women with PCOS.

**Methods:**

A total of 4503 FET cycles that satisfied the inclusion criteria were enrolled in this retrospective cohort study between 2015 and 2020. The GnRH-a group received GnRH-a pretreatment while the AC-FET group did not. Propensity score matching (PSM) method and multivariate logistic regression analysis were performed to adjust for potential confounding factors.

**Results:**

After PSM, women in the GnRH-a group suffered a significantly lower miscarriage rate (11.2% vs. 17.1%, *P* = 0.033) and a higher live birth rate (LBR) compared with those in the AC-FET group (63.1% vs. 56.8%, *P* = 0.043). No differences were observed in the rates of biochemical pregnancy, clinical pregnancy and ectopic pregnancy between the two groups. A higher mean gestational age at birth was observed in the GnRH-a group than in the AC-FET group (39.80 ± 2.01 vs. 38.17 ± 2.13, *P* = 0.009). The incidence of neonatal preterm birth (PTB) in the GnRH-a group was lower than that in the AC-FET group (7.4% vs. 14.9%, *P* = 0.009). Singleton newborns conceived after GnRH-a group were more likely to be small for gestational age (SGA) than those born after AC-FET group (16.4% vs. 6.8%, *P* = 0.009). However, no significant differences were found between the two groups in terms of mean birthweight, apgar score, the rates of macrosomia, large for gestational age and low birth weight.

**Conclusion(s):**

In women with PCOS who underwent AC-FET, GnRH-a pretreatment was significantly associated with a higher live birth rate and a reduced risk of neonatal PTB. However, there was a concomitant increase in the risk of developing SGA babies.

## Background

Polycystic ovary syndrome (PCOS) is one of the most common endocrine disorders in women of reproductive age, accounting for about 70% of anovulatory infertility [1]. Among the strategies for the treatment of infertile PCOS women, frozen embryo transfer (FET) may achieve a higher live birth rate and reduce the risk of ovarian hyperstimulation syndrome (OHSS) compared with fresh embryo transfer [2]. Hence, the application of FET has been recommended as a relatively effective and safer treatment method for this group of infertility patients [3].

Multiple endometrial preparation (EP) cycle protocols have been designed to provide an optimal endometrial environment for embryo implantation in a FET program [4]. However, there is limited consensus on the best means of EP in terms of the reproductive outcomes in women with PCOS [5]. Since PCOS is associated with ovulation dysfunction and irregular menstrual cycles, the most appropriate and frequently used cycle protocol is the artificial cycle FET (AC-FET) [6]. In AC-FET, the endometrium is artificially prepared through consecutive administration of exogenous estrogen and progesterone with or without gonadotropin-releasing hormone agonist (GnRH-a) pretreatment to simulate the natural endocrine environment of the endometrium [7].

GnRH-a is a gonadotropin-releasing hormone (GnRH) analogue with high affinity for pituitary GnRH receptors. After administration, GnRH-a binds to pituitary GnRH receptors and transiently inhibits the hypothalamic–pituitary–gonadal axis, inducing a hypo-estrogenic state [8]. Lower estrogen levels after down-regulation could prevent spontaneous ovulation and prolong the opening period of the “implantation window” to a certain extent [9, 10]. This might be beneficial to the pregnancy outcomes for women undergoing FET.

In assisted reproductive technology (ART), GnRH-a pretreatment combined with AC-FET was found to improve the live birth rate in patients with endometriosis and adenomyosis [11, 12]. However, there is insufficient evidence to indicate whether routine GnRH-a pretreatment should be performed in women with PCOS [13]. Several studies that sought to address this issue were limited by relatively small sample sizes and yielded discordant conclusions [14–16]. Another problem is that the available studies used live birth as the main outcome indicator when evaluating the efficacy and advantages of GnRH-a pretreatment combined with AC-FET, with little consideration for neonatal outcomes after FET. Therefore, whether GnRH-a pretreatment could enhance successful neonatal outcomes in PCOS women remains unknown and need to be further elucidated. In this current study, we aimed to compare the pregnancy and neonatal outcomes of AC-FET with or without GnRH-a pretreatment in women with PCOS.

## Methods

### Study design and participants

This retrospective cohort study was performed at the Xinan Gynecological Hospital in Sichuan, China. Infertile PCOS patients who had received AC-FET with or without GnRH-a pretreatment between 1st January 2015 and 31st December 2020 were enrolled in this study. Patients were diagnosed with PCOS when they had at least two of the following three characteristics according to the Rotterdam criteria [17]: (1) oligo-anovulation or anovulation; (2) clinical and/or biochemical hyperandrogenism; (3) polycystic ovarian morphology on ultrasound. Additionally, patients with any of the following conditions were excluded from the study: other causes of hyperandrogenism and ovulation dysfunction; congenital or acquired uterine malformation; endometriosis and adenomyosis; intrauterine adhesion; a history of recurrent miscarriage; FET cycles after preimplantation genetic testing (PGT) and preimplantation genetic screening (PGS). This study was approved by the Ethics Committee of Chongqing Medical University.

### Endometrial preparation procedures

In the AC-FET protocols, on the 2nd or 3rd day of the menstrual cycle, the patients underwent transvaginal ultrasound examination and basal hormonal level assessment. When the results of both tests indicated that the ovaries were in a basal state (i.e., E2 < 183.5 pmol/L, endometrial thickness < 5 mm, and no pregnancy was present), endometrial preparation was initiated with oral estradiol valerate (2 mg, three times daily; Abbott, Holland). After consecutive administration for 10–12 days, a transvaginal ultrasound scan was carried out to monitor the endometrial thickness and blood test for serum progesterone were performed. If the endometrial thickness was below 7 mm, the dosage of estrogen was increased and the medication duration extended appropriately. If endometrial thickness was still insufficient after 20 days of estrogen administration, the cycle was cancelled. When the endometrial thickness reached ≥ 7 mm, intramuscular injection of progesterone (60 mg/d; Zhejiang Xianju), vaginal administration of Crinone (90 mg/d; Merck, Germany) was commenced.

In the GnRH-a pretreatment group, on the 2–3 day of the menstrual cycle, the patients received a depot of long-acting GnRH agonist (3.75 mg; Diphereline, Ipsen Pharma Biotech, France) intramuscularly. 28 days later, an ultrasound scan and blood testing were performed to confirm complete pituitary down-regulation (that is, E_2_ < 183.5 pmol/L, FSH < 5 U/L, LH < 5 U/L, and endometrial thickness < 5 mm). After meeting the criteria for downregulation, we prepared the endometrium by using the same artificial cycle protocol described above.

All embryos were cryopreserved by the conventional vitrification technique after fresh cycles. The cleavage-stage embryos and blastocysts were thawed on the 4th or 6th day after administration of progesterone, respectively. Embryo transfer was performed under the guidance of transabdominal ultrasound on the day of embryo thawing. In all FET cycles, a maximum of two embryos were transferred per patient. In both the GnRH-a pretreatment group and the AC-FET group, once pregnancy was achieved, luteal support was continued until the 10th to 12th week of gestation.

### Data collection and pregnancy confirmation

Serum hCG level was measured 14 days after embryo transfer to determine the existence of biochemical pregnancy. Clinical pregnancy was confirmed by transvaginal ultrasound examination 26 ~ 30 days after embryo transfer. After confirming clinical pregnancy, special medical staff followed up the patients to obtain their delivery outcomes. The neonatal outcomes data were collected through telephone interviews with the parents after delivery. These data were recorded in the electronic medical records of each patient.

### Outcome measures

The pregnancy and neonatal outcomes of the PCOS women were assessed. The pregnancy outcome measured included biochemical pregnancy rate, clinical pregnancy rate, ectopic pregnancy rate, miscarriage rate and live birth rate. Biochemical pregnancy was defined as serum β-hCG levels > 25 IU/L at 14 days after ET. Clinical pregnancy was defined as the observation of at least one gestational sac in the uterine cavity by ultrasound at 4 weeks after ET. Miscarriage was defined as a loss of clinical pregnancy before the 24th gestational week. Live birth was defined as at least one liveborn baby after 24 weeks of gestation. Primary neonatal outcome measures for singleton infants were as follows: (1) birthweight and its relative outcomes, macrosomia (> 4000 g) and low birthweight (LBW, < 2500 g); (2) gestational age (GA) at birth and preterm birth (PTB, gestational age of < 37 weeks); (3) small for gestational age (SGA) and large for gestational age (LGA). SGA and LGA refer to a birthweight below the 10th percentile and greater than the 90th percentile for the specific gestational age at birth, respectively. The reference of birthweight for gestational age was based on the latest available publications of the national neonatal birthweight curve in China [18].

### Statistical analysis

The version 26.0 of the SPSS software and R package were used for all statistical analyses. Since the selection of EP protocols was not randomized in clinical practice, multiple maternal factors were regarded as potential confounding factors that might regulate the associations between EP protocols and pregnancy-related outcomes. To adjust for such confounding factors, we performed propensity score matching (PSM). PSM is a useful tool to reduce the effects of confounding bias existing in observational studies, which is superior to the conventional method based on regression [19]. Conditional on the PSM, the distribution of baseline covariates measured between the two cohorts is similar, thus simulating the random assignment of subjects in randomized controlled trials [20]. The variables in the PSM included maternal age, BMI, infertility type, endometrial thickness, number of embryos transferred, embryo stage at transfer, number of excellent embryos transferred, and antral follicle count. PCOS women who underwent AC-FET with or without GnRH-a pretreatment were randomly matched in a 1:4 ratio by using the nearest neighbor matching method. The caliper was set to 0.1.

The Shapiro–Wilk test was used to determine whether the continuous data were normally distributed or not. Continuous variables were presented as mean (standard deviation) or median (interquartile range) depending on the distribution characteristics, and categorical variables were presented as numbers (percentages). Before and after PSM, all baseline characteristics and outcome parameters were compared between the two endometrial preparation groups by using Student’s t-test or Mann–Whitney U tests for continuous data, and the Chi-square test or Fisher’s exact test for categorical data, as appropriate.

Women with singleton live births after matching were selected to analyze the association between the type of EP and neonatal outcomes of singletons. As the comparability between the two sets of data was diminished in this process, multivariate logistic regression models were performed to adjust for potential confounders and to calculate the odds ratios (ORs) and the 95% confidence intervals (CIs) for each outcome indicator. A *P*-value < 0.05 (two-tailed) indicated statistically significant differences between groups.

## Results

**Fig. 1:**
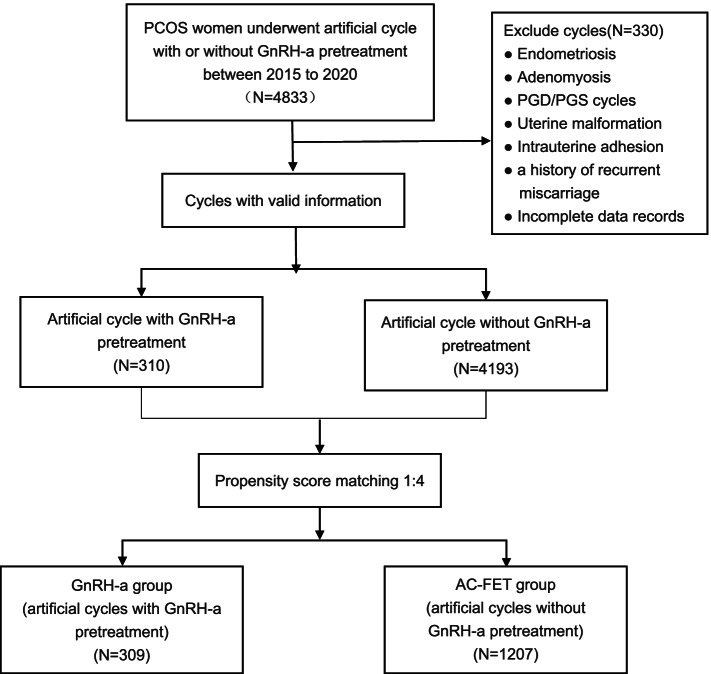
Patient inclusion flowchart

A flowchart of the patient inclusion and exclusion process is shown in Fig. 1. Before matching, a total of 4503 cycles that met the inclusion criteria were included in this study. 310 of these cycles received GnRH-a pretreatment prior to AC-FET. After PSM, 309 of the cycles that received GnRH-a pretreatment were successfully matched to 1207 routine AC-FET.

### Baseline characteristics

Table 1 shows the patient’s baseline characteristics of the two groups before and after matching. Before PSM, the GnRH-a group and the AC-FET group had different baseline characteristics. Women in the GnRH-a group were older (30.11 ± 3.76 vs. 29.17 ± 3.56, *P* < 0.001), had a greater endometrial thickness (9.79 ± 1.77 vs. 9.32 ± 1.66, *P* < 0.001) and a lower level of antral follicle count (27.57 ± 9.66 vs. 29.92 ± 8.90, *P* < 0.001) than those in the AC-FET group. Women in the GnRH-a group experienced more FET cycles and had a lower proportion of first embryo transfer than those in the AC-FET group (58.7% vs. 71.2%, *P* < 0.001). The proportion of blastocysts transferred in the AC-FET group was higher than that in the GnRH-a group (86.7% vs. 82.6%, *P* = 0.042). As expected, the distribution of all baseline characteristics between the two groups were no longer statistically different after the PSM, thereby creating two comparable cohorts that had highly similar characteristics.

**Table 1:** Comparison of baseline characteristics between the two groups before and after PSM

Characteristic	Before PSM			After PSM		
	GnRH-a (*n* = 310)	AC-FET (*n* = 4193)	*P-*value	GnRH-a (*n* = 309)	AC-FET (*n* = 1207)	*P-*value
Maternal age (years)	30.11 (3.76)	29.17 (3.56)	< 0.001	30.07 (3.69)	29.93 (3.62)	0.564
Number of FET cycles			< 0.001			0.755
1	182 (58.7)	2987 (71.2)		181 (58.6)	735 (60.9)	
2–3	118 (38.1)	1141 (27.2)		118 (38.2)	434 (36.0)	
> 3	10 (3.2)	65 (1.6)		10 (3.1)	38 (3.1)	
BMI (kg/m^2^)	23.09 (3.66)	23.19 (3.61)	0.639	23.08 (3.66)	23.02 (3.42)	0.783
Infertility type			0.451			0.696
Primary	197 (63.5)	2753 (65.7)		196 (63.4)	780 (64.6)	
Secondary	113 (36.5)	1440 (34.3)		113 (36.6)	427 (35.4)	
Endometrial thickness(mm) on the day before ET	9.79 (1.77)	9.32 (1.66)	< 0.001	9.77 (1.74)	9.78 (1.75)	0.778
Embryo stage at transfer			0.042			0.779
Cleavage stage	54 (17.4)	558 (13.3)		53 (17.2)	199 (16.5)	
Blastocyst	256 (82.6)	3635 (86.7)		256 (82.8)	1008 (83.8)	
Number of embryos transferred			0.402			0.734
1	55 (17.7)	826 (19.7)		55 (17.8)	205 (17.0)	
2	255 (82.3)	3367 (80.3)		254 (82.2)	1002 (83.0)	
Number of high-quality embryos transferred			0.104			0.722
0	72 (23.2)	1188 (28.3)		71 (23.0)	299 (24.8)	
1	93 (30.0)	1260 (30.1)		93 (30.1)	370 (30.7)	
2	145 (46.8)	1745 (41.6)		145 (46.9)	538 (44.6)	
Baseline hormonal profile at the beginning of AC-FET						
FSH (IU/l)	6.44 (1.89)	6.37 (1.70)	0.504	6.43 (1.89)	6.40 (1.70)	0.792
LH (IU/l)	6.86 (4.23–10.35)	7.20 (4.54–11.45)	0.182	6.83 (4.23–10.31)	6.89 (4.36–10.60)	0.800
E2 (pg/mL)	49.00 (35.00–63.00)	49 (37.00–63.00)	0.695	49 (35.00–63.00)	48 (36–61)	0.610
P(ng/mL)	0.61 (0.40–0.96)	0.62 (0.41–0.93)	0.659	0.61 (0.40–0.96)	0.58 (0.40–0.88)	0.547
AFC	27.57 (9.66)	29.92 (8.90)	< 0.001	27.63 (9.62)	27.83 (8.46)	0.724
AMH (ng/mL)	9.82 (5.63)	10.30 (5.00)	0.110	9.84 (5.64)	9.89 (4.70)	0.764
Number of oocytes retrieved	18.85 (8.60)	19.76 (8.51)	0.069	18.87 (8.60)	18.76 (8.08)	0.833

Data are presented as mean ± SD or median (IQR) for continuous variables and n (%) for categorical variables. *BMI* body mass index, *FSH* follicle-stimulating hormone, *LH* luteinizing hormone, *E2* estradiol, *P* progesterone, *AMH* anti-Mullerian hormone, *AFC* antral follicle count, *PSM* propensity score matching

### Pregnancy outcomes

The comparison of pregnancy outcomes between the two groups before and after PSM is presented in Table 2. No differences were observed in the rates of biochemical pregnancy, clinical pregnancy and ectopic pregnancy between the groups of patients that had the GnRH-a pretreatment and those that did not (*P* > 0.05), either before or after PSM. Nevertheless, in contrast to the routine AC-FET group, women in the GnRH-a group suffered a lower miscarriage rate (11.2% vs. 17.1%, *P* = 0.033), especially early miscarriage rate (6.7% vs. 12.9%, *P* = 0.01). Likewise, the LBR per cycle was higher in the GnRH-a group than in the AC-FET group (63.1% vs. 56.8%, *P* = 0.043).

**Table 2:** Comparison of pregnancy outcomes between the two groups before and after PSM

Outcome	Before PSM			After PSM		
	GnRH-a (*n* = 310)	AC-FET (*n* = 4193)	*P-*value	GnRH-a (*n* = 309)	AC-FET (*n* = 1207)	*P-*value
Biochemical pregnancy rate	242/310 (78.1)	3190 (76.1)	0.428	241/309 (78.0)	924/1207 (76.6)	0.592
Clinical pregnancy rate	224/310 (72.3)	2857 (68.1)	0.132	223/309 (72.2)	837/1207 (69.3)	0.334
Miscarriage rate	26/224 (11.6)	495/2857 (17.3)	0.028	25/223 (11.2)	143/837 (17.1)	0.033
Early miscarriage	16/224 (7.1)	360/2857 (12.6)	0.016	15/223 (6.7)	108/837 (12.9)	0.01
Late miscarriage	10/224 (4.5)	135/2857 (4.7)	0.859	10/223 (4.5)	35/837 (4.2)	0.842
Ectopic pregnancy rate	4/224 (1.8)	38 (1.3)	0.543	4/223 (1.8)	8/837 (1.0)	0.293
Live birth rate	195/310 (62.9)	2321 (55.4)	0.01	195/309 (63.1)	685/1207 (56.8)	0.043

Data are presented as n/total (%)

### Singleton neonatal outcomes

We further compared the neonatal outcomes of singleton infants between the two groups. As shown in Table 3, 122 cycles in the GnRH-a pretreatment group and 424 cycles in the AC-FET group met the criteria for further analysis. Newborns conceived after GnRH-a pretreatment combined with AC-FET had a higher mean GA at birth than those conceived after routine AC- FET (38.80 ± 2.01 vs. 38.17 ± 2.13, *P* = 0.009).

**Table 3:** Comparison of singleton neonatal outcomes between the two groups before and after PSM

Outcome	Before PSM			After PSM		
	GnRH-a (*n* = 122)	AC-FET (*n* = 1464)	*P-*value	GnRH-a (*n* = 122)	AC-FET (*n* = 424)	*P-*value
PTB (< 37 weeks)	9 (7.4)	254 (17.3)	0.004	9 (7.4)	63 (14.9)	0.031
Gestational age (week)	38.80 (2.01)	38.19 (2.34)	< 0.001	38.80 (2.01)	38.17 (2.13)	0.009
Birth weight (kg)	3.27 (0.58)	3.27 (0.62)	0.339	3.27 (0.58)	3.28 (0.58)	0.904
Macrosomia (> 4000 g)	7 (5.7)	103 (6.6)	0.493	7 (5.7)	29 (6.8)	0.666
LBW (< 2500 g)	7 (5.7)	120 (7.7)	0.630	7 (5.7)	31 (7.3)	0.547
Apgar score	9.71 (0.66)	9.66 (0.79)	0.553	9.71 (0.66)	9.68 (0.75)	0.711
LGA	17 (13.9)	311 (21.2)	0.055	17 (13.9)	86 (20.3)	0.142
SGA	20 (16.4)	90 (6.1)	< 0.001	20 (16.4)	29 (6.8)	0.002

Data are presented as mean ± SD, median (IQR) and n (%). *PTB* preterm birth, *LBW* low birthweight, *SGA* small for gestational age, *LGA* large for gestational age

The incidence of PTB in the GnRH-a group was 7.4%, which was lower than that in the AC-FET group (14.9%) (*P* = 0.031). The rate of newborns being SGA also differed between the GnRH-a group and the AC-FET group (16.4% vs. 6.8%, *P* = 0.002). However, no significant differences were found between the two groups in terms of mean birthweight, apgar score, the rates of macrosomia, LGA and LBW(*P* > 0.05).

Multivariate logistic regression models further demonstrated that GnRH-a pretreatment had a significant effect on the neonatal outcomes of women with PCOS (Table 4). Compared to the AC-FET cohort, the aORs for PTB and SGA in the GnRH-a cohort were found to be 0.45 (95% CI: 0.22–0.96, *P* = 0.037) and 2.50 (95%CI: 1.26–4.95, *P* = 0.009), respectively, after adjusting for potential confounders including maternal factors and fetal sex. These were consistent with the results obtained from the univariate analysis.

**Table 4:** Unadjusted and adjusted odds ratios (ORs) of singleton neonatal outcomes in the two groups

Outcomes	Unadjusted OR (95% CI)	*P-*value	Adjusted OR (95% CI)	*P-*value
PTB (< 37 weeks)	0.46 (0.22–0.95)	0.035	0.45 (0.22–0.96)	0.037
Macrosomia (> 4000 g)	0.83 (0.35–1.94)	0.666	0.91 (0.38–2.17)	0.827
LBW (< 2500 g)	0.77 (0.33–1.80)	0.548	0.77 (0.33–1.80)	0.542
LGA	0.63 (0.33–1.18)	0.142	0.64 (0.33–1.22)	0.174
SGA	2.67 (1.45–4.92)	0.002	2.50 (1.26–4.95)	0.009

ORs and 95% CI were based on the univariate analysis. Adjusted ORs and 95% CI were based on the multivariate logistic regression model after adjusting for age, BMI, number of FET cycles, infertility type, endometrial thickness, number of embryos transferred, embryo stage at transfer, number of high-quality embryos transferred

## Discussion

Although FET has been widely used in women with PCOS to improve reproductive outcomes and reduce the risk of OHSS, the optimal cycle regimen for this population is yet to be determined [21]. In this present retrospective study, we found that GnRH-a pretreatment was associated with a reduced miscarriage rate and a higher LBR following AC-FET in women with PCOS. In addition, a significantly lower rate of PTB and a higher rate of SGA were observed in the GnRH-a group as compared to the routine AC-FET group.

### Pregnancy outcomes

The effect of GnRH-a pretreatment on pregnancy outcomes in PCOS women receiving AC-FET has not been fully clarified. A retrospective study, which exclusively focused on hyperandrogenic PCOS women [14], reported that pretreatment with GnRH-a significantly increased the ongoing pregnancy rate, possibly by reducing the level of serum androgens. Another retrospective study evaluated the clinical outcomes of GnRH-a combined with artificial cycle in five groups of infertile women, and indicated that GnRH-a was effective in improving the LBR for all types of infertility tested, especially for women with PCOS(15). In contrast to these findings, a more recent randomized controlled trial showed that GnRH-a pretreatment did not improve LBR for women with PCOS who received AC-FET, but significantly increased the cost of treatment for these patients(16). Therefore, the authors posited that AC-FET without GnRH-a pretreatment may be a better choice for women with PCOS. However, the effects of the two EP methods on neonatal outcomes are unclear.

In this present study, which involved a larger sample size and adjustment for important confounding factors, we observed that PCOS women who received GnRH-a pretreatment prior to AC-FET had a higher LBR and a lower risk of miscarriage. One possible mechanism for this effect is that the use of GnRH-a might have improved endometrial receptivity by counteracting the hyperandrogenic status of the PCOS patients. Hyperandrogenism is one of the main manifestations of metabolic disorders in PCOS patients. It has been proposed that hyperandrogenism may have adverse effects on pregnancy outcomes in several levels of the ART process, such as reducing endometrial receptivity [22, 23] and affecting the quality of oocytes /embryos [24, 25]. Elevated levels of free androgen index could interfere with the development of the endometrium, leading to a significant increase in the risk of miscarriage in subsequent pregnancies [26]. By inhibiting the hypothalamus-pituitary-ovary (HPO) axis, GnRH-a is able to decrease estrogen and androgen levels, which may contribute to the success of FET [27].

Another possible mechanism is that GnRH-a pretreatment prevents the impairment of endometrial receptivity caused by high levels of LH. The ratio of serum FSH/LH in patients with PCOS is usually inverted. High levels of LH could act on progesterone receptors in the endometrium, leading to premature endometrial transition from proliferative phase to secretory phase. This is not conducive to the synchronization of endometrial development and embryo implantation [28, 29].

### Neonatal outcomes

Previous observational studies have shown that different EP protocols have different impacts on perinatal outcomes of the general infertile population, implying that the cycle regimens may be an independent factor related to fetal growth after FET [30]. Our study has reported the effect of GnRH-a pretreatment combined with AC-FET on neonatal outcomes among singletons born to PCOS women, and showed that GnRH-a pretreatment was independently associated with a reduced risk of PTB and an increased risk of SGA in newborns of PCOS women.

Apart from the fact that PTB is a significant cause of mortality in neonates and children under 5 years old, it is also related to the increased risk of neurodevelopmental disorders and chronic diseases in adulthood [31]. Therefore, considering the adverse effects of PTB on the short- and long-term health of newborns, a timely prediction of the risk of PTB is critical for early clinical intervention. Several studies have shown that both the diagnosis of PCOS and artificial cycle regimens are independent risk factors for PTB in newborns conceived through ART [32–35]. Christ et al. [36] reported that among the three major characteristics of PCOS, hyperandrogenism, rather than polycystic ovarian morphology or oligo-anovulation, was associated with PTB. In PCOS women without hyperandrogenism, Hu et al. found that there was no increase in the incidence of abnormal neonatal outcomes compared with non-PCOS patients [37]. However, if pretreatment with ethinyl estradiol/cyproterone acetate is performed to inhibit the effect of androgens on PCOS women, the risk of newborns being PTB would be reduced [38]. Based on these reports, we speculate that the use of a GnRH-a and subsequent androgen deprivation might be an important reason for the reduced risk of neonatal PTB observed in this study. Therefore, different phenotypes and characteristics of PCOS patients could serve as indicators to enhance an appropriate EP protocol selection by physicians.

The birthweight of a newborn is mainly determined by GA at birth and the growth rate of the fetus. In the present study, although singleton infants in the GnRH-a group had a longer duration of gestation than those conceived through the routine AC-FET, we found that there was no significant difference in mean birthweight and the rates of LBW/macrosomia between the two groups. Furthermore, singleton infants born after AC-FET with GnRH-a pretreatment were more likely to be SGA. It is well known that in the whole population, the birthweight of the fetus is basically normally distributed, with lower odds of developing SGA(the left tail of the birthweight curve) and LGA(the right tail of the birthweight curve). We speculate that GnRH-a pretreatment prolong the duration of gestation in PCOS women and retard fetal weight gain in a modest but significant way, thereby increasing the risk of SGA. This could be understood that the birthweight curve for the same GA was presented as a relatively smooth positive skewness distribution in the GnRH-a group [30]. However, further studies are needed to validate these findings and clarify the underlying mechanisms.

### Strengths and limitations

To the best of our knowledge, this is the largest population study in this field to assess pregnancy-related outcomes following AC-FET with or without GnRH-a pretreatment in women with PCOS. Unlike previous studies that mainly focused on live birth, our study also provided more complete information on the effectiveness and safety of GnRH-a pretreatment by analyzing the neonatal outcomes in PCOS women. Additionally, our study included extensive control for potential confounding differences between GnRH-a and the routine AC-FET groups via PSM and multivariate logistic regression models, thus creating two similar cohorts.

We acknowledge that in this study, we did not obtain information about pregnancy complications, such as gestational hypertension, preeclampsia, and gestational diabetes, which are potential risk factors for adverse neonatal outcomes [39, 40]. This may have a confounding effect on the results. Another limitation is that the neonatal data were obtained through telephone follow-up,which might have led to some information bias.

## Conclusions

Our study demonstrated that in women with PCOS who underwent AC-FET, GnRH-a pretreatment was significantly associated with an increase in LBR and a reduced risk of neonatal PTB. However, the incidence of newborns being SGA was also significantly increased at the same time. Therefore, before applying the GnRH-a pretreatment regimen in PCOS women, it seems necessary to take some measures to reduce the risk of neonatal SGA events. Further studies are needed to verify our findings and clarify the underlying mechanisms.

## Data Availability

The datasets used and/or analysed during the current study are available from the corresponding author on reasonable request.

## Abbreviations

FET: Frozen embryo transfer
GnRH-a: Gonadotropin-releasing hormone agonist
PCOS: Polycystic ovary syndrome
AC-FET: Artificial cycle FET
PSM: Propensity score matching
LBR: Live birth rate
GA: Gestational age
LBW: Low birthweight
PTB: Preterm birth
SGA: Small for gestational age
LGA: Large for gestational age
OHSS: Ovarian hyperstimulation syndrome
EP: Endometrial preparation
ART: Assisted reproductive technology
PGT: Preimplantation genetic testing
PGS: Preimplantation genetic screening
E2: Estradiol
FSH: Follicle-stimulating hormone
LH: Luteinizing hormone
hCG: Human chorionic gonadotropin
OR: Odds ratio
CI: Confidence interval
BMI: Body mass index
HPO: Hypothalamus-pituitary-ovary
P: Progesterone
AFC: Antral follicle count
AMH: Anti-Mullerian hormone
SD: Standard deviation
IQR: Inter-quartile range
